# Stem cell-paved biobridge facilitates neural repair in traumatic brain injury

**DOI:** 10.3389/fnsys.2014.00116

**Published:** 2014-06-24

**Authors:** Naoki Tajiri, Kelsey Duncan, Alesia Antoine, Mibel Pabon, Sandra A. Acosta, Ike de la Pena, Diana G. Hernadez-Ontiveros, Kazutaka Shinozuka, Hiroto Ishikawa, Yuji Kaneko, Ernest Yankee, Michael McGrogan, Casey Case, Cesar V. Borlongan

**Affiliations:** ^1^Center of Excellence for Aging and Brain Repair, Department of Neurosurgery and Brain Repair, Morsani College of Medicine, University of South FloridaTampa, FL, USA; ^2^Sanbio Inc., Mountain ViewCA, USA

**Keywords:** trauma, cell transplantation, regenerative medicine, neurogenesis, extracellular matrix

## Abstract

Modified mesenchymal stromal cells (MSCs) display a unique mechanism of action during the repair phase of traumatic brain injury by exhibiting the ability to build a biobridge between the neurogenic niche and the site of injury. Immunohistochemistry and laser capture assay have visualized this biobridge in the area between the neurogenic subventricular zone and the injured cortex. This biobridge expresses high levels of extracellular matrix metalloproteinases (MMPs), which are initially co-localized with a stream of transplanted MSCs, but later this region contains only few to non-detectable grafts and becomes overgrown by newly recruited host cells. We have reported that long-distance migration of host cells from the neurogenic niche to the injured brain site can be attained via these transplanted stem cell-paved biobridges, which serve as a key regenerative process for the initiation of endogenous repair mechanisms. Thus, far the two major schools of discipline in stem cell repair mechanisms support the idea of “cell replacement” and the bystander effects of “trophic factor secretion.” Our novel observation of stem cell-paved biobridges as pathways for directed migration of host cells from neurogenic niche toward the injured brain site adds another mode of action underlying stem cell therapy. More in-depth investigations on graft-host interaction will likely aid translational research focused on advancing this stem cell-paved biobridge from its current place, as an equally potent repair mechanism as cell replacement and trophic factor secretion, into a new treatment strategy for traumatic brain injury and other neurological disorders.

## A novel brain repair mechanism of transplanted stem cells

**Figure 1:**
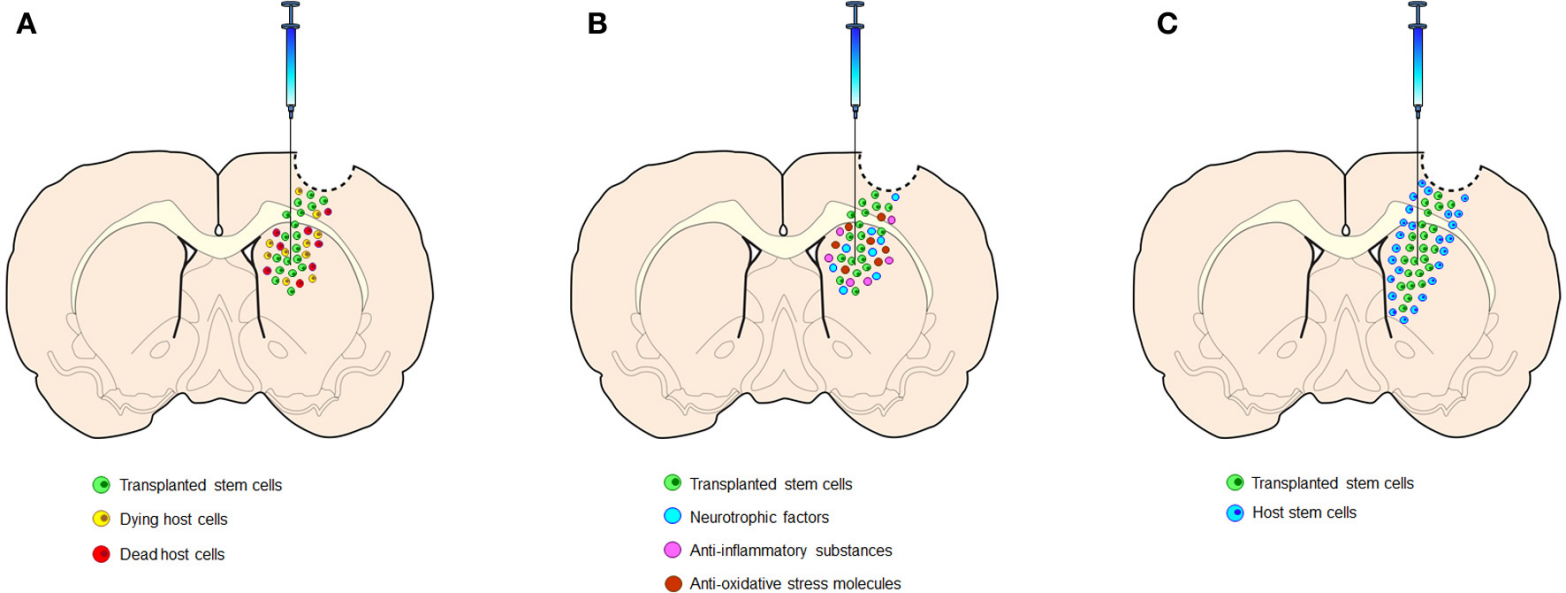
**Multiple mechanisms underlying stem cell therapy. (A)** Cell replacement entails that transplanted cells replace dead or dying host cells, which would require neuronal differentiation of stem cells and reconstruction of the damaged synaptic circuitry. **(B)** By-stander effects involve secretion of neurotrophic factors, anti-inflammatory substances, and anti-oxidative stress molecules by the transplanted cells, which subsequently rescue spared host cells or stimulate neurogenesis. **(C)** Stem cell-paved biobridges show that transplanted cells form a biological pathway, enriched in MMPs, and ferries newly born host stem cells from the neurogenic niche SVZ to the injured host tissue.

Stem cell research offers an avenue for an in-depth study of cell biology, as well as in the development of new strategies to treat diseases (Yasuhara et al., 2006, 2008; Tajiri et al., 2012). Nevertheless, much remains to be understood about the mechanisms underlying the beneficial effects of stem cell therapy. To date, there are two major schools of discipline in stem cell-mediated repair mechanism in brain damage caused by injury or neurodegenerative disorders (Borlongan et al., 2004; Pastori et al., 2008). The first concept supports the idea of “cell replacement,” i.e., stem cells implanted into the brain directly replace dead or dying cells (Figure 1A), whereas the other argues that transplanted stem cells secrete growth factors that indirectly rescue the injured tissue (i.e., bystander effects of stem cells) (Lee et al., 2007; Redmond et al., 2007) (Figure 1B).

Stem cells exist through adulthood (Ma et al., 2010), and possess the capacity for self-renewal and differentiation into multiple lineages. They have also been shown to contribute to the maintenance of homeostasis (Kim et al., 2011), exert therapeutic benefits both endogenously (Barha et al., 2011; Borlongan, 2011; Jaskelioff et al., 2011; Wang et al., 2011) and following transplantation into injured organs, e.g., the brain (Mazzocchi-Jones et al., 2009; Hargus et al., 2010; Lee et al., 2010; Andres et al., 2011; Liu et al., 2011; Mezey, 2011; Yasuda et al., 2011). There are two major stem cell niches in the brain namely, the subventricular zone (SVZ) of the lateral ventricles and the subgranular zone of the hippocampus dentate gyrus (DG) (Carlén et al., 2009; Sanai et al., 2011), although quiescent neural stem cells (NSCs) have been found in other brain regions (Robel et al., 2011). The discovery that stem cells are activated following injury opened up new research frontiers in regenerative medicine (Yasuhara et al., 2006; Mazzocchi-Jones et al., 2009; Hargus et al., 2010; Lee et al., 2010; Andres et al., 2011; Barha et al., 2011; Borlongan, 2011; Jaskelioff et al., 2011; Liu et al., 2011; Mezey, 2011; Wang et al., 2011; Yasuda et al., 2011; Tajiri et al., 2012). Consequently, this research paved the way for translation of laboratory studies on stem cells into limited clinical trials for brain disorders (Pollock et al., 2006; Yasuhara et al., 2009; Seol et al., 2011). Despite these scientific advances and clinical applications, the mechanisms underlying stem cell-mediated repair in brain injury are not yet completely understood.

In a recent study, we demonstrated motor and neurological improvements in rats subjected to traumatic brain injury (TBI) and transplanted intracerebrally with cultured Notch-induced human bone marrow-derived mesenchymal stromal cells (referred to as SB623, supplied by SanBio Inc.) While we obtained important results that corroborate the putative therapeutic benefits of stem cell transplantation for TBI, our research on the mechanism of action of SB623 revealed breakthrough findings which support the discovery of a novel stem-cell mediated repair mechanism in brain injury. Accordingly, we observed the capacity of transplanted stem cells to harness a “biobridge” between the neurogenic niche and the site of brain injury, enabling long-distance migration of host neurogenic cells and consequently, initiating endogenous repair mechanisms. In this paper, we discuss the properties and characteristics of these stem cell paved-biobridges, elaborate on the unique mechanism by which these biobridges facilitate repair in a rat model of TBI, and importantly, suggest the clinical significance of exploiting this novel stem cell-mediated concept of brain repair for the treatment of brain injury and other neurological disorders.

## Breakthrough discovery: stem cell-paved formation of “biobridges” in experimental models of TBI

Rats subjected to TBI were intracerebrally transplanted with SB623 (gene-modified human mesenchymal stromal cells) (Zhao et al., 2007; Yasuhara et al., 2009). Thereafter, the motor and neurological functions of these rats were evaluated at 1, 2, and 3 months post-TBI, and histological studies were performed to assess the therapeutic effects of SB623 transplantation. The behavioral studies showed significant motor and neurological improvement in TBI rats which received SB623. Histological studies also showed profound reduction in TBI-induced damages to the cortical core and the peri-injured cortical areas in SB623-transplanted TBI rats. The behavioral and histological improvements, however, were achieved despite minimal graft survival—0.60 and 0.16% at 1 and 3 months, respectively. These findings led us to examine the condition of the host tissue, in view of functional recovery despite lack of graft persistence.

At 1 month post-TBI, we observed notable increases in endogenous cellular proliferation (Ki67) as well as immature neural differentiation (nestin) in the peri-injured cortical areas and SVZ, along with a stream of migrating cells along the corpus callosum (CC) of SB623 transplanted TBI animals. Furthermore, at 3 months post-TBI, we observed enhanced cellular proliferation and neural differentiation in the peri-injured cortical (CTX) areas of SB623 transplanted TBI animals, accompanied by a solid stream of neuronally-labeled cells (nestin and DCX) migrating not only along but also across the CC from the SVZ to the impacted CTX. Contrastingly, TBI rats which received vehicle infusion exhibited elevated cellular proliferation; however the newly formed cells were “trapped” within the SVZ and CTX and did not migrate to the impacted CTX.

We next analyzed the formation of the biobridge by means of the cells leaving the SVZ and moving toward the site of injury was laser captured in the animals receiving the SB623 cells.

The biobridge between the SVZ and the impacted cortex was composed of highly proliferative, neutrally committed, and migratory cells. These animals showed a 2 and 9-fold upregulation of the matrix metalloproteinase-9 (MMP) activity and expression at 1 and 3 months post-TBI, respectively. Further *in vitro* studies also showed the ability of SB623 cells to enhance cell migration via this MMP-rich signaling cues. These signals are crucial to the migration of endogenous cells which can then assist with functional recovery of damaged tissue. Merely 1 month post-TBI, a surge of proliferative Ki67 positive cells and neurally immature nestin labeled cells in the peri-injured areas and SVZ were discerned. The high level of MMP-9 in the biobridge indicates the importance of this neurovascular proteinase. Interestingly, this proteinase was upregulated in the vehicle group, but reverted back to control-sham levels at 3 months post-TBI. This illustrates the role of MMP in long-term recovery and adds another facet to the mechanism through which stem cells aid in recovery of damaged tissue.

To provide further evidence that the implanted SB623 cells facilitated the formation of the biobridge, thus enabling the migration of host stem cells from the SVZ to the site of injury and the up-regulation of endogenous cells, an *in vitro* study was performed with primary rat cortical cells grown both alone and co-cultured with SB623 cells. These were grown either in the presence or absence of the MMP-9 inhibitor Cyclosporin-A. Migratory cell assay revealed noticeably enhanced migration of primary rat cortical cells in the chamber containing SB623, which was then significantly suppressed by treatment with the MMP-9 inhibitor. Treatment with the inhibitor alone, combined treatment with SB623 and the inhibitor, and absence of both SB623 and the inhibitor did not significantly alter migratory potential.

Although endogenous repair mechanisms are initiated post-TBI, these effects are typically limited to the neurogenic SVZ and quiescent neurogenic resident cells around the impacted cortex. Accordingly, these endogenous mechanisms are not robust enough to provide a solid defense against TBI or other disease-induced cell death cascades necessitating introduction of exogenous cells to aid migration of endogenous stem cells from the neurogenic niche to the site of injury. Stem cell transplantation into the peri-injured cortical areas purportedly creates a biobridge comprised of a neurovascular matrix which allows newly formed endogenous cells to migrate efficiently to the site of injury. Moreover, biobridge is established, exogenous cells slowly fade away, supplanted by newly formed endogenous cells that can maintain recovery even in the absence of transplanted stem cells.

## A biobridge between the neurogenic niche and the ischemic tissue

The results show SB623 transplants aid in regeneration of the traumatically injured brain by constructing a biobridge between the SVZ and the peri-injured cortex (Figure 1C). This novel mechanism opens new doors for cell therapy by allowing the creation of similar biobridges between neurogenic and non-neurogenic sites to aid in injury-specific migration of cells across tissues that are barriers to cellular motility.

A phase I/IIa study of SB623 cell transplantation in chronic stroke patients has already been initiated. Transplantation of SB623 cells has been shown to mitigate histological and behavioral deficits associated with stroke, spinal cord injury, and Parkinson's Disease in both cell culture and animal models of brain disorders. The use of SB623 cells in TBI patients is an innovative concept that has already been FDA approved for a limited clinical trial based on the data presented.

Understanding SB623's role in facilitating the migration of endogenous cells via a biobridge exposes the active role of MMPs and ECMs in stroke pathology (Park et al., 2009; el Zoppo et al., 2012) and highlights their increasingly prominent role as therapeutic targets for stroke. Functions and levels of MMPs and ECMs have been shown to be influenced by a variety of cells coming from variable sources including umbilical cord blood, peripheral blood, and the adult brain (Barkho et al., 2008; Sobrino et al., 2012; Lin et al., 2013). This suggests a potential for these molecules to serve as biobridges similar to the current function of Notch-induced SB623 mesenchymal stromal cells.

While documented neurogenic niches such as the SVZ exist in the adult brain and contain cells that play a critical role in repairing the stroke brain (Ekdahl et al., 2009; Ducruet et al., 2012; Hassani et al., 2012; Wang et al., 2012; Trueman et al., 2013), it has been shown that a major limiting factor for endogenous repair is the lack of successful migration of newly formed host cells to the site of injury. Currently, results show SB623 cell transplantation boosts endogenous repair mechanisms by guiding the transition of new cells from the neurogenic SVZ, across a non-neurogenic brain area, to the site of injury. The ability of SB623 cells to form biobridges containing MMPs and ECMs which can then entice newly formed cells from the niche into the ischemic tissue appears to be a fundamental mechanism of action. Once the transplanted SB623 cells have pioneered the formation of these biobridges, they allow the endogenous stem cells to take over the remodeling process.

The precise mechanism by which the graft cells are integrated into the recipient brain tissue and subsequently interact with the host cells to result in functional restoration remains unknown. This essential interaction between the transplanted cell and host cell becomes even more obscure when graft survival is minimal. This indicates that the role of the SB623 is to set in motion robust and stable therapeutic benefits, particularly by leading the way for host cells to reach the injured site even across non-neurogenic and injured tissues. MMP has been implicated in chronic brain injury recovery through studies that inhibited MMPs, which consequently halted neurogenic migration from the SVZ into damaged tissues. The result was a noticeable retardation in neurovascular remodeling. This supports the concept that exogenously added cells can express MMPs and thereby reinforce the neurovascular unit, aiding in the transplant-mediated host cell migration toward the site of injury. This potentially allows for the formation of biobridges, thereby affording functional recovery in TBI.

## Beyond TBI: exploiting stem cell-paved biobridges for the treatment of other neurological disorders

The most adult stem cells in the brain are found in the SVZ of the lateral ventricles and the subgranular zone of the hippocampus dentate gyrus. The microenvironment of a stem cell niche is maintained by the signaling molecules, growth factors, and receptors. In adults, these stem cells typically remain in a dormant non-dividing state until activated by an insult. When an insult occurs motility ensues and the endogenous stem cells find themselves trapped and unable to reach the site of injury. The novel discovery that grafted cells can facilitate endogenous stem cell migration from the neurogenic niche to the site of injury is indeed a significant progress in the both stem cell and TBI research.

Our recent study (Tajiri et al., 2013) advances the concept of the biobridge mechanism as a cell mediated repair strategy in TBI, and opens new avenues for translational applications of cell therapy in TBI. Monitoring long term safety and efficacy of SB623 cell therapy in TBI animal models in order to optimize the conduct of clinical trials involving these cells in TBI patients is a priority. Gaining a more concrete understanding of the stem cell mechanism is the next step which will help to push forth the boundaries of stem cell research and explain the mechanism of cellular therapy in neurological disease.

In addition to TBI, a number of other neurological disorders are characterized by a “biological gap” between the site of injury and intact tissue. In this regard, disorders like stroke and Parkinson's disease may benefit from a deeper understanding on the concept of stem cell-paved biobridge because both disorders present with a site of cellular degeneration that is physically separated from the area of the brain that could aid in the recovery of dead tissue or lost cells. For example, stroke entails an ischemic core and penumbra residing next to intact tissue. While the ischemic core damage cannot be recovered, the potential for neural repair has been demonstrated by targeting the penumbra. Accordingly, a biobridge between the penumbra and the intact tissue (i.e., neurogenic niche) could potentially aid in stroke. Parkinson's disease involves the degeneration of the nigrostriatal dopaminergic pathway, which could improve drastically with directed migration of host stem cells toward this region in the form of a biobridge. Further investigations are warranted to determine how the concept of stem-cell paved biobridge could be exploited for the treatment of other neurological disorders.

## Multi-pronged mechanisms underlying stem cell therapy

As noted above, stem cell-mediated repair mechanisms have been widely purported as afforded via cell replacement and bystander effects (Snyder et al., 1997; Borlongan et al., 2004; Lee et al., 2007; Redmond et al., 2007; Pastori et al., 2008; Acosta et al., 2014). Our proposed third mechanism describes the recruitment of endogenous stem cells from the stem cell niches (Alvarez-Buylla et al., 2008) to the injured site through the transplanted stem cell-formed biobridge. This begs the question of whether the biobridge provides the scaffold or trophic factors to promote stem cell migration. Interestingly, new reports suggest candidate ECMs may serve as a scaffold or trophic factor-rich soluble molecules. For example, a study observed that the limits of interstitial cell migration depends upon scaffold porosity and deformation of the nucleus, with pericellular collagenolysis and mechanocoupling as modulators acting as scaffolds and assisting with biobridge formation (i.e., stem cell migration) (Wolf et al., 2013). Alternatively, a study found that functional analysis of mesenchymal stem cell proliferation, migration, and adhesion to ECMs revealed that IL-1β did not affect proliferation but served to induce the secretion of trophic factors and adhesion to ECM components such as collagen and laminin (Carrero et al., 2012).

In the end, stem cell functionality is a key factor to achieve clinically relevant outcomes of stem cell therapy. Of note, neurogenesis *per se* does not equate to these new neurons integrating with the damaged area, and that physiological and functional assays (e.g., synaptic circuitry reconstruction, evoked potentials, long-term potentiation, etc.) will be necessary to unequivocally reveal the contribution of these newly formed cells in the resulting behavioral recovery. Depending on the target disease of stem cell therapy, it is likely that neuronal differentiation to specific disease-phenotype (Hong et al., 2011), as in the case of Parkinson's disease and Huntington's disease, may be needed to produce functional effects. However, we caution that such neuronal differentiation may occur in both exogenously transplanted cells and the mobilized endogenous stem cells. Our concept of biobridge formation highlights the need for these stem cells to be guided toward the site of injury, which may facilitate the therapeutic effects of these newly formed cells that have committed to neuronally differentiated cells. Alternatively or a complement mechanism in the absence of neuronal differentiation, we also propose the biobridge-facilitated by-stander effects, in that with the directed migration of these stem cells toward the site of injury, the biobridge is able to harness the secretion of growth factors, anti-inflammatory substances, and/or anti-oxidative stress molecules close to the damaged area.

The concept of stem cell-paved biobridge may have some similarities with the use of olfactory ensheathing glia in spinal cord injury. Compelling evidence from animal models and clinical studies suggest that transplantation of olfactory ensheathing cells (OECs), specialized glia in the olfactory system, combined with specific training may be therapeutically useful in central nervous system (CNS) injuries and neurodegenerative diseases. The unique function of OECs could be attributed to both production of cell adhesion molecules and secretion of growth factors in OECs, which support neuron survival and neurite outgrowth (He et al., 2013). Another study showed that transplantation of OEG and Schwann cell (SCs) in a sub-acute phase can improve anatomical outcomes after a contusion injury to the spinal cord by increasing the number of spared/regenerated supraspinal fibers, reducing cavitation, and enhancing tissue integrity (Barbour et al., 2013). Most spinal cord injury models evaluating the therapeutic efficacy of OEC transplants have reported functional recovery via indirect and direct reparative pathways involving growth factor secretion, neuronal and axonal regeneration, and remyelination (Roet et al., 2013).

Although similarities exist between the biobridge seen with OECs in spinal cord injury and our observed biobridge in TBI, the biobridge seen in spinal cord injury involves the ensheating features of OECs, as well as the fabrication of scaffolds (such as laminin and fibronectin) and seeding the stem cells onto these matrices in order to create a biobridge. In contrast, our observed biobridge involves a natural process of the stem cells themselves serving as matrices in facilitating the migration of endogenous stem cells from the neurogenic niche toward the injured host tissue. With this in mind, a similar biobridge strategy was documented in Parkinson's disease whereby the transplanted dopamine-secreting cells were deposited along the nigrostriatal system (instead of merely transplanting the cells into the striatum) to closely reconstruct the major dopaminergic afferent and efferent pathways (Wang et al., 1996; Tang et al., 1998; Chiang et al., 2001). Compared to our observed biobridge formation, this bridging graft in Parkinson's disease is an artificial reconstruction of the dopaminergic system whereby micro-deposits of immature cells are undertaken along the nigrostriatal pathway, whereas our biobridge formation reveals a natural process of the transplanted stem cells homing from the neurogenic niche and forming a bridge toward the injured site, and subsequently attracting endogenous stem cells to populate the biobridge and to eventually continue the brain reparative process.

Altogether, these observations suggest that while distinct mechanisms of action may be individually facilitating the neural repair of transplanted stem cells in the injured brain, overlapping regenerative processes involving cell replacement, by-stander effects, and biobridge formation may be working in tandem in realizing the therapeutic benefits of stem cell therapy.

### Conflict of interest statement

Cesar V. Borlongan is an inventor on a patent application related to the stem cell research reported here. Cesar V. Borlongan received research financial support from SanBio Inc. for this study. Cesar V. Borlongan is additionally supported by NIH NINDS R01NS071956-01, James and Esther King Foundation for Biomedical Research Program, Celgene Cellular Therapeutics, KMPHC and NeuralStem Inc.
